# Psychotherapy employed additionally to Psychopharmacotherapy is not related to Better Treatment Outcome in Major Depressive Disorder

**DOI:** 10.1192/j.eurpsy.2022.229

**Published:** 2022-09-01

**Authors:** L. Bartova, G. Fugger, M. Dold, M. Mitschek, J. Zohar, J. Mendlewicz, D. Souery, S. Montgomery, C. Fabbri, A. Serretti, S. Kasper

**Affiliations:** 1 Medical University of Vienna, Department Of Psychiatry And Psychotherapy, Vienna, Austria; 2 Chaim Sheba Medical Center, Psychiatric Division, Tel Hashomer, Israel; 3 Free University of Brussels, School Of Medicine, Brussels, Belgium; 4 Psy Pluriel, Centre Européen De Psychologie Médicale, Brussels, Belgium; 5 University of London, Imperial College School Of Medicine, London, United Kingdom; 6 University of Bologna, Department Of Biomedical And Neuromotor Sciences, Bologna, Italy

**Keywords:** antidepressant treatment, Psychotherapy, Psychopharmacotherapy, major depressive disorder

## Abstract

**Introduction:**

Although numerous effective antidepressant (AD) strategies are available for the treatment of major depressive disorder (MDD), many patients do not achieve satisfactory treatment response.

**Objectives:**

The aims of the present European, cross-sectional, multicenter, naturalistic study were (1) to determine the proportion of patients suffering from primary MDD who received additional psychotherapy to their ongoing psychopharmacotherapy and (2) to identify the associated socio-demographic and clinical patterns.

**Methods:**

Patients receiving both treatments were compared to those lacking concomitant additional psychotherapy that was manual-driven psychotherapy (MDP) in all cases.

**Results:**

While 68.8% of a total of 1279 MDD patients received exclusively psychopharmacotherapy, 31.2% underwent a psychopharmacotherapy-MDP combination. The latter patient population was rather younger, higher educated, employed, exhibited an earlier mean age of MDD onset, lower severity of current depressive symptoms with lower odds of suicidality and higher rates of melancholic features, and comorbid asthma and migraine, and was generally treated with lower daily doses of their first-line ADs. Whereas agomelatine was more commonly dispensed in these patients, selective serotonin reuptake inhibitors were more often prescribed in MDD patients lacking additional MDP. No significant between-group differences were detected in terms of treatment outcome.

**Conclusions:**

The fact that the employment of additional MDP was not related to better treatment outcome in MDD represents our major and clinically most relevant finding. Generally, MDP was employed in a minority of our patients who experienced rather beneficial socio-demographic and clinical characteristics. This might reflect an inferior accessibility of these psychotherapeutic techniques for patients who are more severely ill and less socio-economically privileged.

**Disclosure:**

References Bartova L, Fugger G, Dold M, Swoboda MMM, Zohar J, Mendlewicz J, Souery D, Montgomery S, Fabbri C, Serretti A, Kasper S. Combining psychopharmacotherapy and psychotherapy is not associated with better treatment outcome in major depressive disor

